# Nonlinearity and sex differences in the performance of a polygenic risk score for juvenile idiopathic arthritis

**DOI:** 10.3389/fimmu.2025.1531390

**Published:** 2025-05-22

**Authors:** Kristine Løkås Haftorn, Hamid Khoshfekr Rudsari, Piotr Pawel Jaholkowski, Vilde Øverlien Dåstøl, Sigrid Valen Hestetun, Ole A. Andreassen, Clarice R. Weinberg, Helga Sanner

**Affiliations:** ^1^ Department of Rheumatology, Oslo University Hospital, Oslo, Norway; ^2^ Center for Precision Psychiatry, Division of Mental Health and Addiction, Oslo University Hospital, and Institute of Clinical Medicine, University of Oslo, Oslo, Norway; ^3^ K. G. Jebsen Centre for Neurodevelopmental Disorders, University of Oslo and Oslo University Hospital, Oslo, Norway; ^4^ Biostatistics and Computational Biology Branch, National Institute of Environmental Health Sciences, National Institutes of Health, Bethesda, MD, United States; ^5^ Norway and Institute of Clinical Medicine, University of Oslo, Oslo, Norway

**Keywords:** juvenile idiopathic arthritis, polygenic risk score, nonlinearity, sex differences, gene-sex interaction

## Abstract

**Background:**

Juvenile idiopathic arthritis (JIA) is an immune-mediated pediatric disease believed to result from a complex interplay of genetic and environmental factors. Genome-wide association studies have enabled calculation of polygenic risk scores (PRS) for JIA. Understanding how the PRS associates with JIA and whether it performs similarly across sexes is essential for its utility in future studies.

**Methods:**

We studied the relationship between a PRS developed from a previously published genome-wide association study of JIA and JIA in children from the Norwegian Mother, Father and Child Cohort Study (MoBa; total n = 57,630; JIA cases = 238). Generalized linear models (GLM) and generalized additive models (GAM) were used in logistic regression to assess the association. Furthermore, we investigated whether the relationship between PRS and JIA differed by sex by applying GAM models with interaction terms.

**Results:**

PRS was significantly associated with JIA using both GLM (p< 2e-16) and GAM (p< 2e-16) models, and our results indicated a nonlinear relationship between PRS and JIA (effective degrees of freedom, EDF = 1.96). We found a significant interaction between sex and JIA PRS in relation to JIA (p = 0.017), and indications of a stronger and more logit-nonlinear relationship in females (EDF = 1.82) versus males (EDF = 1.06).

**Conclusion:**

The relationship between PRS and JIA was slightly logit-nonlinear for females and logit-linear for males. The PRS for JIA can likely be used either as a continuous or discrete variable in analyses, but sex-stratification is recommended for future studies.

## 1 Introduction

Juvenile idiopathic arthritis (JIA) is an immune-mediated disease characterized by joint inflammation lasting for at least six weeks and presenting before the age of 16 (1). It is a heterogenous disease with seven subtypes, and it is more prevalent in girls (2, 3). JIA imposes a significant burden on patients, their families, and society. It is believed to result from a complex interplay of genetic and environmental factors, although causal factors and underlying mechanisms remain largely unknown (4).

Familial, twin, and genome-wide association studies (GWAS) have helped to approach and dissect the genetic contribution to complex diseases, including JIA (5, 6). The monozygotic twin concordance rate of JIA has been estimated as 25-40%, and the sibling recurrence risk ratio as 11.6 (1). In the so far largest GWAS of JIA, including 3305 cases and 9196 controls, López-Isac et al. identified numerous susceptibility loci for JIA with a total SNP-based heritability of 0.61 (7).

The results from GWAS studies can be exploited by constructing polygenic risk scores (PRS), comprising aggregated effects of variants across the genome, which can be used to estimate the individual’s genetic risk for the outcome of interest (8). PRS have been widely applied in studies of a range of different diseases and phenotypes and can be particularly useful in studies assessing the relationship between genetic and environmental risk factors for disease (9). Although PRSs have been suggested as potential clinical tools in the future, there are several obstacles that need to be addressed before they can be implemented into a clinical setting (9). PRSs are therefore so far mainly useful as research tools for studying genetic risk.

Recently, we developed a PRS for the children in the Norwegian Mother, Father and Child cohort study (MoBa) based on results from the aforementioned GWAS by López-Isac et al. (7, 10). When including a PRS in statistical models, either as a main effect or interaction variable, it is important to know how it relates to the outcome, in our case JIA. Understanding how the risk of JIA changes depending on the PRS can inform whether the PRS can be used as a continuous variable in the model or if it should be grouped into a discrete variable, and if so, how the discrete variable should be defined (11). Traditional logistic regression assumes a linear relationship between predictors and the log-odds of the outcome. However, some biological associations, including those between genetic risk scores and disease, may not follow a strictly linear pattern. Using nonlinear methods for modelling can therefore be useful because they are flexible enough to capture more complex relationships between the PRS and JIA. Furthermore, the PRS may be performing differently in specific subgroups, such as males and females, which can also be important to uncover when including the PRS in studies of risk and disease development (12).

Sex-specific genetic associations appear to play a role in a number of autoimmune and immune-mediated diseases, but the degree to which these differences contribute to JIA susceptibility has not been fully studied (13). A recent study on JIA patients found that the presence of antinuclear antibodies (ANA) was associated with specific genes, and this was observed more frequently in females, suggesting an interaction between certain genes and sex (14). Furthermore, a female-specific association between the *PTPN22* SNP rs2476601 and JIA has been confirmed across several different populations (15, 16), and evidence of a sex-specific association of *PSMA6/PSMC6/PSMA3* genetic variants with subtypes of JIA has also been reported (17). However, genome-wide studies of JIA, including the GWAS on which our PRS is based, were not stratified by sex (7). To address potential sex differences, it is thus important to assess whether the PRS performs similarly in males and females.

To fill these knowledge gaps, our aims of this study were 1) to investigate the relationship between the PRS for JIA and the probability of a JIA diagnosis, and 2) to explore whether the relationship between the PRS and JIA risk is different between males and females.

## 2 Methods

### 2.1 Study population and design

MoBa is a large-scale pregnancy cohort study led by the Norwegian Institute of Public Health (NIPH), which recruited participants across Norway between 1999 and 2008. 41% of the eligible women participated. The cohort comprises around 114,500 children, 95,200 mothers, and 75,200 fathers (18, 19). The present study uses version 12 of the MoBa data files, which underwent quality assurance and were made available for research in January 2019. We included MoBa children who had previously been genotyped (20).

### 2.2 Outcome

Information about JIA status was collected by linkage to the Norwegian Patient Registry (NPR), which includes data with personal ID numbers from all Norwegian public hospitals and specialists with public funding from 2008 (21). In Norway, the university hospitals with specialists within pediatric rheumatology have the main responsibility of diagnosing and following JIA patients. Cases were born between 1999 and 2009 and diagnosed with JIA before December 2021. We defined a JIA case as having at least two International Classification of Diseases (ICD)-10 codes (≥2 M08, ≥2 M09, or ≥1 M08 *and* ≥1 M09). In a recent validation of this case definition, we found a positive predictive value of 93.4% (10), ensuring a low number of false positive diagnoses. It is therefore reasonable to assume that our case definition largely reflects accurate diagnoses. For cases who received their first ICD-10 code in 2021, we accepted a single relevant ICD-10 code (M08 or M09), as we received our latest updates from NPR in December 2021. Controls were defined as non-JIA cases, and we removed all controls who had one ICD-10 code (M08 or M09) because they might have JIA.

### 2.3 Polygenic risk score for JIA

Umbilical cord blood samples were collected at birth, and the extracted DNA was frozen and stored at NIPH. The genotyping, quality control and imputation of the genetics data of the samples in MoBa have been extensively described previously (20). We calculated PRSs from the results of a previously published GWAS of JIA (7) by applying PRSice, version 2.3.3 (22). We chose *p*-value thresholds of 5E-8, 1E-6, 1E-5, 1E-4, 1E-3, 1E-2, 5E-2, 1E-1, and 1 to calculate PRSs and then extracted the first principal component (PC) for PRSs across all the thresholds, using this first PRS-PC as our final PRS for JIA (23). We then, using the whole dataset, standardized the PRS to a mean of zero and a standard deviation (SD) of 1 (24) and we used the standardized PRS for all analyses. In sensitivity analyses, the PRS was categorized into (1) quartiles, forming four equal-sized categories, (2) three categories containing the top 10%, middle 80% and bottom 10% of observations, and (3) a binary variable based on the median (**Supplementary Table 1**).

### 2.4 Statistical analysis

R version 4.2.3 was used to conduct all statistical analyses (25), and all scripts are available in our GitHub repository (https://github.com/KristineLH/PRS-JIA-sex). We used multiple logistic regression and generalized additive models (GAM) to examine the relationship between PRS and JIA. The top 10 PCs from the whole genotype dataset, together with sex, and year of birth were included as covariates in the models.

#### Nonlinear modeling approach

To account for potential logit-nonlinearity, we applied GAM using the *gam* function from the mgcv package (26). GAM extends traditional regression by allowing flexibility in how predictors influence the outcome, fitting smooth, data-driven curves rather than assuming a fixed logit-linear form. In our model, PRS was modeled as a smooth function using a regression spline, which adapts to the shape of the data. The effective degrees of freedom (EDF) from the GAM output served as an indicator of nonlinearity, with an EDF of 1 representing a linear relationship and values greater than 1 suggesting a nonlinear relationship (27).

#### Modeling sex differences

To investigate whether the relationship between the PRS and JIA differed by sex, we first included an interaction term between the PRS and sex in the multiple logistic regression model. The Wald test was used to assess statistical significance of the interaction, and a *p*-value < 0.05 was regarded as significant. However, interaction terms in standard regression models assume a constant, linear modification of the association by sex, which may not fully capture potential differences in the way the PRS is associated with JIA in males and females. To address this, we further investigated sex-specific patterns by fitting separate smooth splines for the PRS in males and females. Specifically, we created new variables by multiplying PRS with dummy variables for each sex and then modeled these products as smooth terms in the GAM framework. This allowed us to estimate the association between the PRS and JIA in each sex separately.

#### Visualization

**Figure 1:**
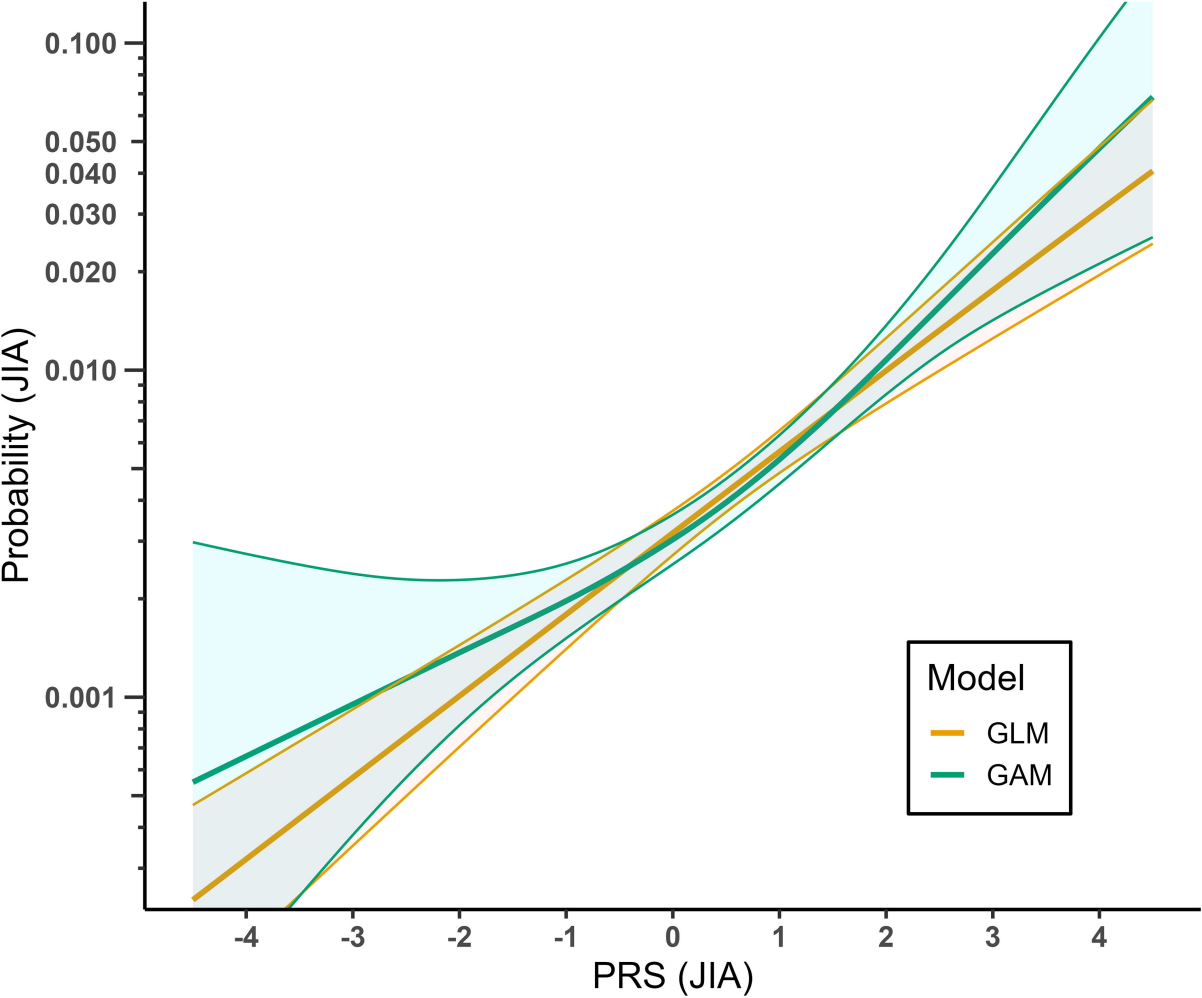
Relationship between PRS for JIA and probability of JIA modelled by a generalized linear model (GLM) compared to a generalized additive model (GAM). The lines show the fitted prediction model of JIA probability ~ PRS + sex + year of birth + top 10 principal components for each of the models. The colored areas represent the 95% confidence intervals for the corresponding models.

**Figure 2:**
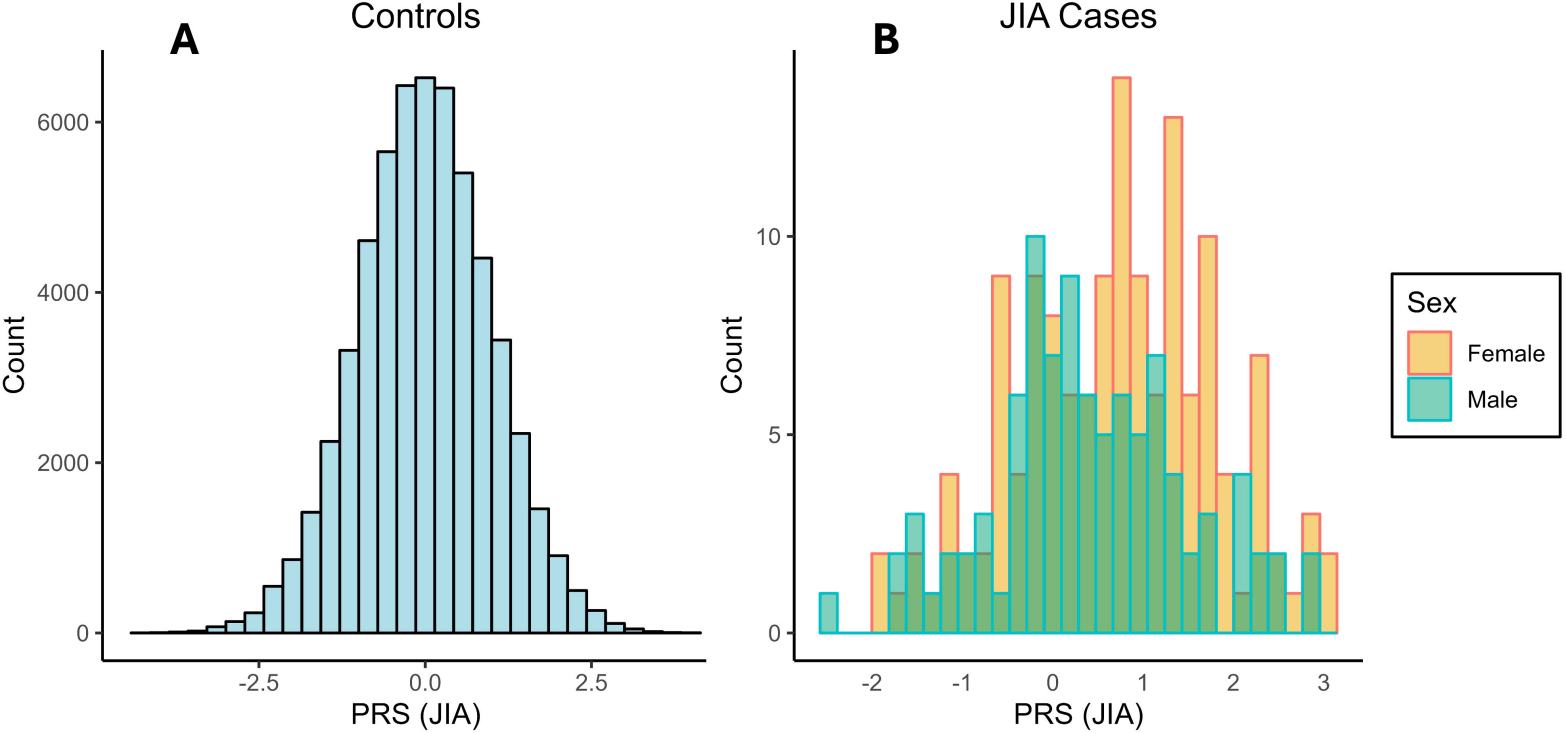
Distribution of JIA PRS in **(A)** controls, and **(B)** JIA cases grouped by sex.

To aid interpretation, we visualized the relationship between PRS and JIA for each model. Using the *predict* function, we calculated the probability of JIA across a range of PRS values (-4.5 to 4.5 with an increment of 0.1), while keeping other covariates (10 PCs, year of birth) at their mean values. This enabled direct comparison of PRS effects across methods (**Figure 1**) and sexes (**Figure 2**).

## 3 Results

### 3.1 Study sample characteristics

Our final analytical sample included 57,630 children of whom 238 were identified as JIA-cases (**Table 1**). Male participants comprised 51.0% (n = 29,139) of the controls, compared to only 39.9% (n = 91) of the JIA cases. The JIA cases had a mean PRS of 0.58 (+/- 1.10 SD), whereas the mean PRS in controls was -0.002 (+/- 1.00 SD).

**Table 1:** Study sample characteristics.

Characteristics	JIA cases			Controls		
	All	Male	Female	All	Male	Female
Sample size (n,%)	238 (100)	95 (39.9)	143 (60.1)	57,392	29,319 (51.0)	28,073 (48.9)
Year of birth (mean, SD)	2005 (2.18)	2004 (2.19)	2005 (2.15)	2005 (2.17)	2005 (2.17)	2005 (2.16)
PRS (mean, SD)	0.579 (1.10)	0.399 (1.08)	0.699 (1.11)	-0.002 (1.00)	0.005 (1.00)	-0.010 (0.99)

### 3.2 Association between PRS and JIA

We assessed the association between PRS and JIA using a standard logistic regression model (GLM) and a generalized additive model (GAM), results shown in **Figure 1**. In both models, PRS was significantly associated with JIA (p < 2e-16 for both models), and the results were similar for the categorized PRS variables (**Supplementary Figure 1**). The EDF in our GAM model was 1.939, indicating a logit-nonlinear relationship between PRS and risk of JIA.

### 3.3 The association between PRS and JIA differs by sex

In **Figure 2**, we show the distributions of PRS in controls, as well as cases stratified by sex. The PRS distributions for controls show a mean of 0.01 in males and -0.01 in females. In contrast, JIA cases demonstrate higher PRS means. Specifically, the PRS mean for male cases is 0.40, while for female cases, it is 0.70, indicating a stronger association between PRS and JIA diagnosis in females compared to males.

**Figure 3:**
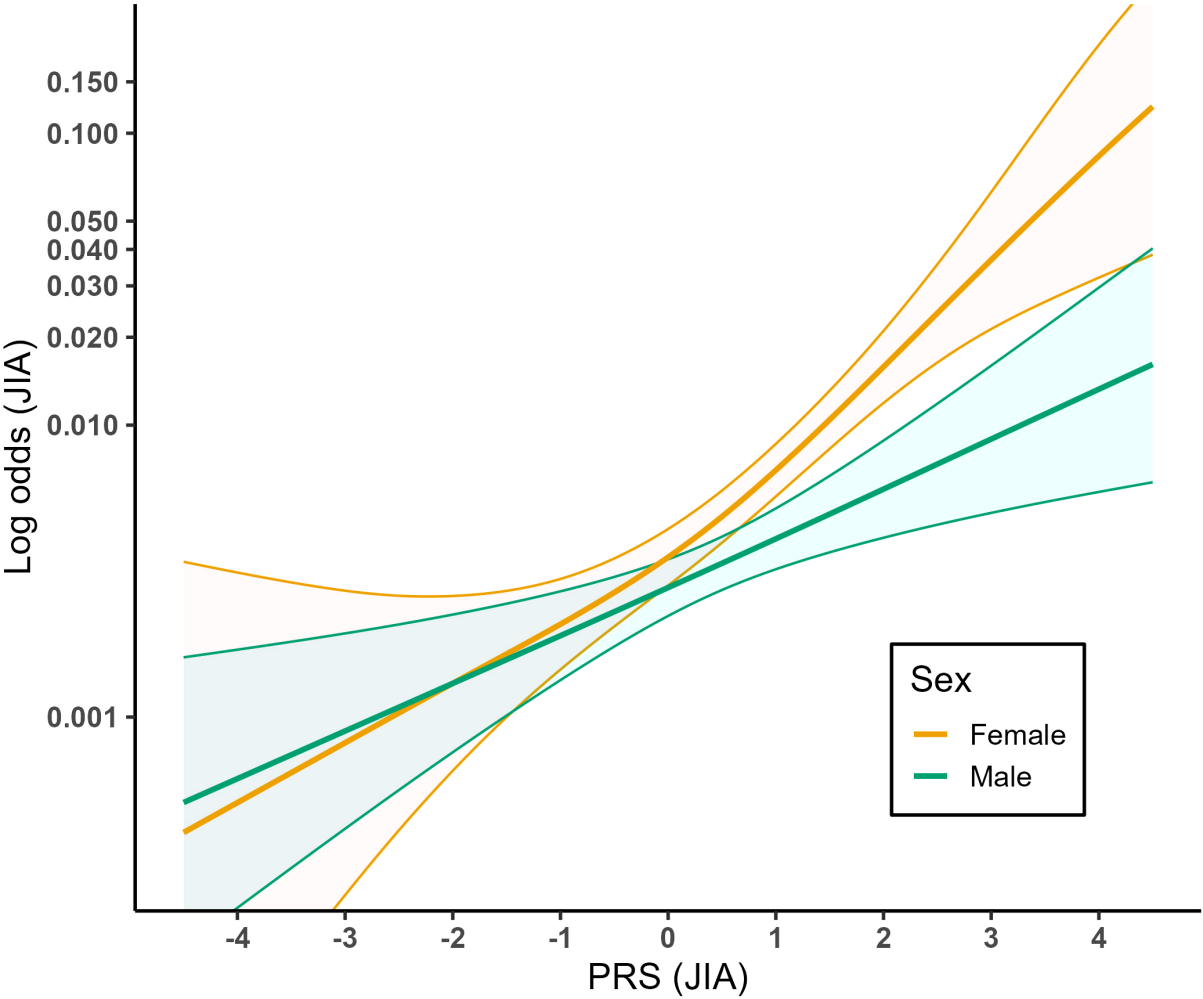
Relationship between PRS of JIA and probability of JIA in females and males. The lines show the fitted prediction model of JIA probability ~ PRS + sex + year of birth + top 10 principal components for each of the sexes. The colored areas represent the 95% confidence intervals for the corresponding models.

We further investigated the interaction between sex and PRS in association with JIA. In a simple logit-linear model, the interaction term between sex and PRS was significantly associated with JIA (p = 0.017). We then investigated this interaction further by conducting a semi-stratified analysis allowing for nonlinear relationships (**Figure 3**). This model showed that PRS was significantly associated with JIA in both females (p < 2e-16) and males (p < 0.001). Interestingly, the relationship between PRS and JIA was approximately logit-linear in males (EDF = 1.06) but showed a larger tendency of logit-nonlinearity in females (EDF = 1.82). We detected a similar pattern when defining the PRS as high- and low-risk variable divided into top 10%, bottom 10% and middle 80% of observations (**Supplementary Figure 2**).

## 4 Discussion

Our results show that the relationship between PRS and JIA is weakly logit-nonlinear. The notable difference in PRS distribution between male and female JIA cases underscores a sex-specific variation in PRS among JIA cases in the MoBa cohort. Furthermore, we show a significant interaction between sex and PRS in relation to JIA, with sex acting as a PRS effect measure modifier. Interestingly, the logit-nonlinearity of the relationship seems to be driven by the females, whereas in males the relationship seems to be logit-linear.

Understanding the relationship between a PRS and the outcome of interest is important when the PRS is to be used in further analyses, such as when investigating interactions between environmental exposures and genetic predisposition to develop JIA. Particularly, for the PRS to be used as a continuous variable in analyses of JIA, the relationship between PRS and JIA should be well modelled (11). A non-linear relationship between the PRS and JIA could suggest that, for risk prediction, the PRS should be grouped into a discrete variable. Our results indicate a somewhat logit-nonlinear relationship between the PRS for JIA and risk of JIA, with a stronger effect with higher PRS compared to lower PRS. However, as shown in **Figure 1**, the GAM model taking logit-nonlinear associations into account is not vastly different from the simple logit-linear model. It should be noted, however, that the relatively small number of JIA cases in our dataset may have limited our power to detect subtle nonlinear interactions, particularly for males. Although grouping the PRS into a categorical variable as shown in **Supplementary Figure 1** gave a similar fit, the predicted probabilities of JIA were lower than with the continuous PRS, especially for the high-risk groups. This indicates some loss of information and shrinkage towards the mean due to grouping the PRS. Thus, we suggest using PRS as a continuous variable in future studies when possible. Grouping the PRS into high- and low-risk groups of top 10%, bottom 10% and middle 80% gave the most similar fit compared to using the PRS as a continuous variable and may therefore be an alternative way of modelling the PRS. However, males and females appear to require distinct models for use of this PRS for JIA.

Sex-specific and sex-dependent effects of PRSs for other diseases, like schizophrenia and coronary artery disease have also been reported (28–30). The difference we observe in PRS performance between the sexes could reflect differences in the sex ratio among cases and controls in the GWAS our PRS is based on (12), with the girl cases outnumbering the boys and consequently having more influence on the formation of the score. However, the sex ratios were not stated in the GWAS paper, which may limit our results (7). Furthermore, different subtypes of JIA are associated with different genetic loci, and sex distribution also differs depending on the subtype (3). Some subtypes, such as oligoarticular and polyarticular JIA, which constitute around 70% of all cases, occur 2–3 times more frequently in girls, but not all JIA subtypes are more common in females (3). Thus, the PRS may be mainly reflecting genetic predisposition for the more common subtypes which are also more common in females and therefore show a stronger association with JIA in females compared to males. We did not have access to information on subtypes in our dataset and were thus not able to account for this in our analyses. Given that certain JIA subtypes differ in their genetic patterns, this represents a limitation of our study. Furthermore, gene-environment interactions involving exposures that differ by sex, such as hormones, have not been accounted for and may have influenced our results. Finally, our results may indicate that the effect of genetic predisposition on JIA development is dependent on biological processes that differ between the sexes.

When using the PRS for JIA in association and interaction analyses, researchers should be aware of the sex-specific associations and consider sex-stratification when possible. Our findings suggest that future studies on the genetic predisposition to JIA, including GWAS and the development of PRS, should incorporate sex-specific analyses to identify genetic loci that may contribute to disease development in males and females separately, as well as those shared between sexes (31, 32). Developing a set of distinct PRS scores specifically for sex-by-subtype categories could prove to be even more usefully predictive, but this would require a very large genetic dataset with detailed information on sex and JIA subtypes. We also suggest exploring potential susceptibility loci for JIA on the X-chromosome (33) as this was not included in our study nor, to our knowledge, in any GWAS of JIA thus far. As sex differences are common in autoimmune diseases in general, investigating sex-specific associations of PRS may be relevant also for other autoimmune and immune-mediated diseases (34).

In conclusion, our results show that the relationship between our PRS and JIA is slightly logit-nonlinear, but only for females. The PRS for JIA can likely be used either as a continuous or discrete variable in analyses, but sex-stratification should be considered. Future studies should further investigate sex-differences in genetic predisposition of JIA and other autoimmune diseases.

## Data Availability

Access to MoBa data can be obtained by applying to the Norwegian Institute of Public Health (NIPH). Restrictions apply regarding the availability of these data, which were used under specific approvals for the current study and therefore not publicly available. Access can only be given after approval by the Regional Committees for Medical and Health Research Ethics (REC) in compliance with the EU General Data Protection Regulation (GDPR) and approval from the data owners. The consent given by the participants does not open for storage of data on an individual level in repositories or journals. Requests to access these datasets should be directed to helsedata.no/en.
