# Supplementary material for: Nonlinearity and sex differences in the performance of a polygenic risk score for juvenile idiopathic arthritis
Source: Front Immunol. 2025 May 22;16:1531390. doi: 10.3389/fimmu.2025.1531390 (PMC12142061; doi:10.3389/fimmu.2025.1531390)
Supplement: Supplementary file 1 [file DataSheet1.docx]

Supplementary Material

# Supplementary Tables

**Supplementary Table 1: PRS categories**

|  | **JIA cases** | | | **Controls** | | |
| --- | --- | --- | --- | --- | --- | --- |
|  | **All** | **Male** | **Female** | **All** | **Male** | **Female** |
| **Total sample size (n)** | 238 | 95 | 143 | 57,392 | 29,319 | 28,073 |
| **PRS quartiles (n, %)** |  |  |  |  |  |  |
| 1 (-4.24 - -0.664) | 28 (11.7) | 14 (14.7) | 14 (9.8) | 14380 (25.1) | 7278 (24.8) | 7102 (25.3) |
| 2 (-0.664 - 0.002) | 48 (20.2) | 22 (23.2) | 26 (18.2) | 14359 (25.0) | 7328 (25.0) | 7031 (25.0) |
| 3 (0.002 - 0.667) | 47 (19.7) | 22 (23.2) | 25 (17.5) | 14360 (25.0) | 7358 (25.1) | 7002 (24.9) |
| 4 (0.667 – 4.03) | 115 (48.3) | 37 (38.9) | 78 (54.5) | 14293 (24.9) | 7355 (25.1) | 6938 (24.7) |
| **PRS bottom/top 10% (n, %)** |  |  |  |  |  |  |
| Bottom 10% | 13 (5.5) | 7 (7.4) | 6 (4.2) | 5750 (10.0) | 2913 (10.0) | 2837 (10.1) |
| Middle 80% | 161 (67.6) | 70 (73.7) | 91 (63.6) | 45943 (80.1) | 23406 (79.8) | 22537 (80.3) |
| Top 10% | 64 (26.9) | 18 (18.9) | 46 (32.2) | 5699 (9.9) | 3000 (10.2) | 2699 (9.6) |
| **PRS binary (n, %)** |  |  |  |  |  |  |
| Low (< -0.002) | 76 (31.9) | 36 (37.9) | 40 (28.0) | 28657 (50.0) | 14553 (49.6) | 14104 (50.2) |
| High (0.002 ≤) | 162 (68.1) | 59 (62.1) | 103 (72.0) | 28735 (50.0) | 14766 (50.4) | 13969 (49.8) |

# Supplementary Figures


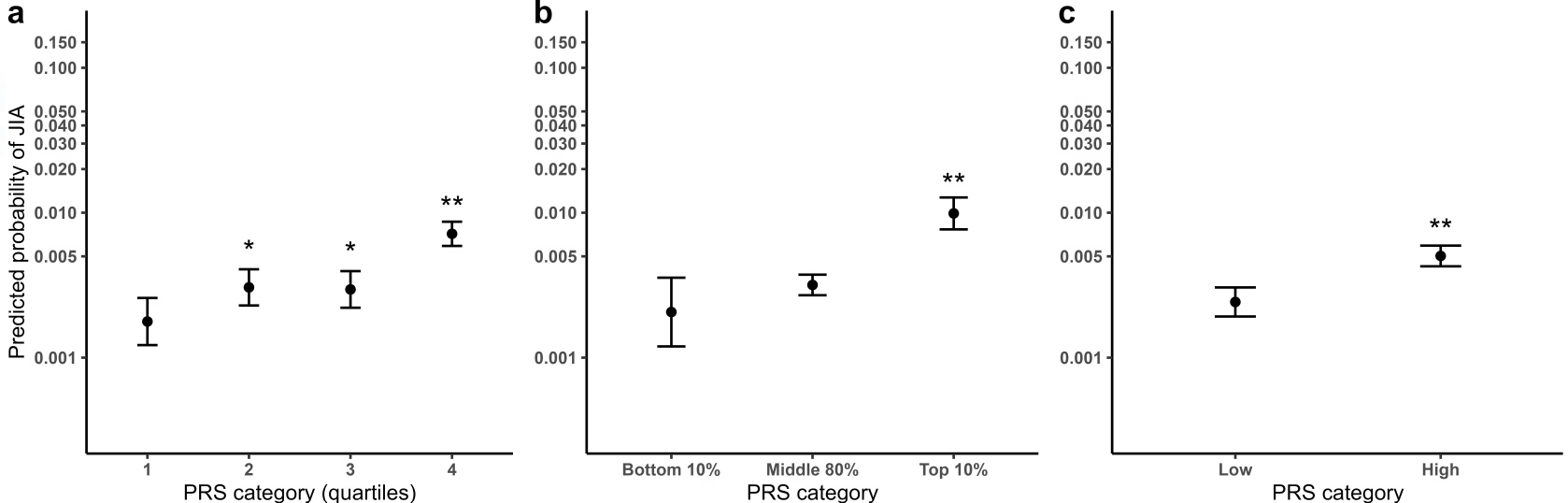


**Supplementary Figure 1.** Relationship between PRS for JIA and predicted probability of JIA modelled by a generalized linear model including sex, year of birth and top 10 principal components as covariates. a) PRS is categorized into quartiles 1-4 with quartile 1 as reference. b) PRS is categorized into the bottom 10% (reference), top 10% and middle 80% of the dataset. c) PRS is categorized into a binary variable where “Low” (reference) include values lower than the median value, whereas “High” include values equal to or higher than the median value. The bars represent the 95% confidence intervals for the probability estimates. * indicates a p value < 0.05, whereas ** indicates a p value < 0.0001.


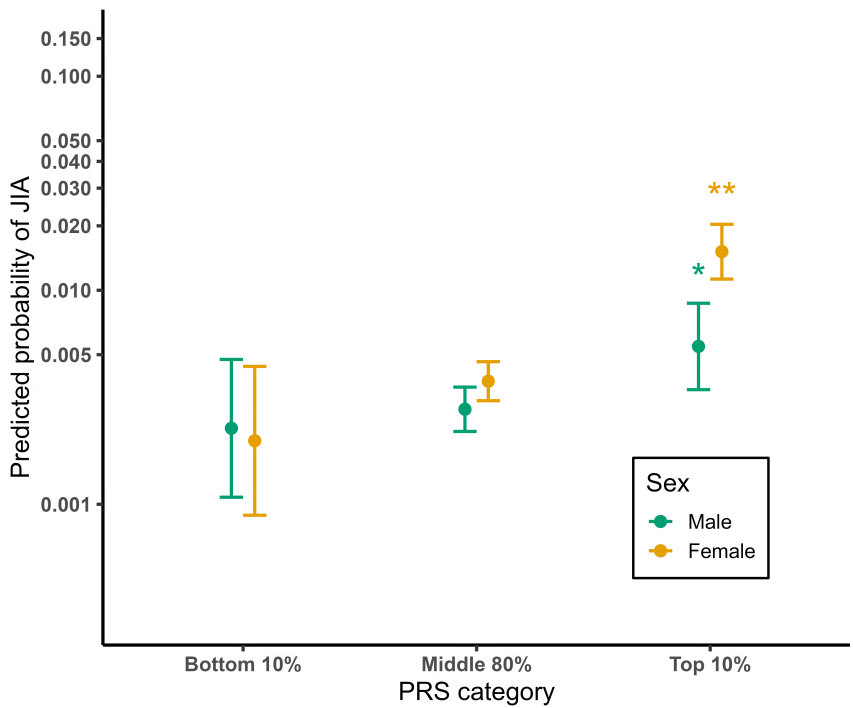


**Supplementary Figure 2.** Relationship between PRS for JIA and predicted probability of JIA in females and males modelled by a generalized linear model including year of birth and top 10 principal components as covariates. PRS is categorized into the bottom 10% (reference), top 10% and middle 80% of the dataset. The bars represent the 95% confidence intervals for the probability estimates. * indicates a p value < 0.05, whereas ** indicates a p value < 0.0001.
